# Optimization of Mechanosensitive Cross-Talk between Matrix Stiffness and Protein Density: Independent Matrix Properties Regulate Spreading Dynamics of Myocytes

**DOI:** 10.3390/cells11132122

**Published:** 2022-07-05

**Authors:** Judith Brock, Julia Erhardt, Stephan A. Eisler, Marcel Hörning

**Affiliations:** 1 Biobased Materials Laboratory, Institute of Biomaterials and Biomolecular Systems, University of Stuttgart, 70569 Stuttgart, Germany; judith.brock@bio.uni-stuttgart.de (J.B.); julia.erhardt@bio.uni-stuttgart.de (J.E.); 2 Stuttgart Research Center Systems Biology (SRCSB), University of Stuttgart, 70569 Stuttgart, Germany; stephan.eisler@srcsb.uni-stuttgart.de

**Keywords:** mechanosensitivity, proliferation, myocyte, actincytoskeleton, extracellular matrix, L-DOPA

## Abstract

Cells actively sense differences in topology, matrix elasticity and protein composition of the extracellular microenvironment and adapt their function and morphology. In this study, we focus on the cross-talk between matrix stiffness and protein coating density that regulates morphology and proliferation dynamics of single myocytes. For this, C2C12 myocytes were monitored on L-DOPA functionalized hydrogels of 22 different elasticity and fibronectin density compositions. Static images were recorded and statistically analyzed to determine morphological differences and to identify the optimized extracellular matrix (ECM). Using that information, selected ECMs were used to study the dynamics before and after cell proliferation by statistical comparison of distinct cell states. We observed a fibronectin-density-independent increase of the projected cell area until 12 kPa. Additionally, changes in fibronectin density led to an area that was optimum at about 2.6 $\mu {g/\mathrm{cm}}^{2}$, which was confirmed by independent F-actin analysis, revealing a maximum actin-filament-to-cell-area ratio of 7.5%. Proliferation evaluation showed an opposite correlation between cell spreading duration and speed to matrix elasticity and protein density, which did not affect cell-cycle duration. In summary, we identified an optimized ECM composition and found that independent matrix properties regulate distinct cell characteristics.

## 1. Introduction

Forces in biology can act at various scales, ranging from entire muscle actuation of larger mammals down to single subcellular actin contraction of individual cells. While muscles are controlled by the nervous system, single cells self-regulate and respond to the extracellular matrix (ECM) through mechanosensitive properties [1]. There are many external cues that enable cells to actively react to the extracellular microenvironment through different signaling pathways. External passive properties of the ECM, i.e., elasticity [2,3] and topology [4,5], are sensed through focal adhesion complexes [6] and can lead to changes in cell morphology and regulative signaling. The latter are large macromolecular assemblies distributed at the plasma membrane of cells that can trigger mechanical force generation via actin–myosin complexes [7]. Variable environmental signals, i.e., galvanotaxis, phototaxis and chemotaxis, can also lead to activation of intracellular force generation. Heart cells, for example, may generate a signaling cascade (membrane potential) to induce cell contraction through an initial increase of sodium concentration through connective gap-junctions of adjacent cells [8]. During development and starvation, *Dicytostelum discoideum* cells sense the excretion of extracellular cAMP and light in order to form multicellular structures [9]. Chemical gradients also play an important role during embryogenesis and morphogenesis in many other species, such as in Drosophila [10], mice [11] and even plants [12].

As cells have the ability to sense and react to the ECM, it is crucial to provide an optimal mechanical, topological and molecular environment in artificial culture systems. Here, the term ‘optimal’ refers to the natural environment, as cells have developed to fulfill a specific function, which is predetermined by the developmental lineage of a specific environment [13]. Specific examples are an optimal matrix stiffness of about 12 kPa that mimics muscle tissue elasticity, leading to optimized myotube differentiation [14], cardiac wave beat [15] and synchronization dynamics [2], as well as actomyosin stress fiber alignment [16]. On the other hand, the optimal matrix protein density (collagen, 0.1–1.0 μg/mL) of the ECM leads to an increased spread of smooth muscle cells [17], and different cell types and matrix proteins may exhibit very different optimal conditions, such as epithelial cells cultured with fibronectin at 0.05 μg/mL [18].

During the last decade, many advanced methods have been developed to study the influence of dynamic ECM changes on cells, such as dynamically tuneable hydrogels [3,19], three-dimensional scaffolds [4,20] and even 3D-printed cell scaffolds that mimic native tissue composition and mechanics [5]. However, the influence and cross-talk between matrix stiffness and protein density has not been studied very conclusively and systematically yet. In this study, we focus on the morphology and proliferation dynamics of single C2C12 muscle cells on rigid acrylamide-based hydrogels that are functionalized with distinct fibronectin densities. By statistical analysis of an extended number of cells, we first reproduced the well-established relationship between projected cell area and substrate rigidity, as shown before for various cell types, e.g., C2C12 [3,14,19], embryonic cardiomyocytes [15], mesenchymal stem cells [13] and epidermal stem cells [21]. We identified a transition from smaller to larger projected cell areas independent of fibronectin density at about 12 kPa. Next, we fixed the substrate rigidity and varied the fibronectin densities, revealing an optimal fibronectin density at about 2.6 $\mu {g/\mathrm{cm}}^{2}$, where cells exhibit not only the largest cell areas but also an optimally situated actin cytoskeleton. Based on these findings, we statistically investigated the proliferation dynamics of cells through parameterization of distinct morphological cell states before and after cell division. Most interestingly, we found that the spreading speed of the cell area depends on fibronectin density, while the duration of spreading after cell division depends solely on matrix rigidity. Although cells displayed distinct mechano–regulative dynamics that depend on ECM composition, they did not show changes in cell-cycle duration.

## 2. Materials and Methods

### 2.1. Glass Preparation

Glass substrates (22 mm, Δh = 0.12–0.17 mm, Matsunami, C022001) and cover glasses ($24\times 24\;{\mathrm{mm}}^{2}$, VWR, ECN631-1571) were cleaned using a modified RCA method [3,22] according to the following procedure. The glasses were (1) subsequently rinsed and sonicated in acetone, ethanol, methanol and distilled water for 3 min; (2) immersed in hydrogen peroxide solution (H${}_{2}$O:H${}_{2}$O${}_{2}$:NH${}_{3}$ aq. as 5:1:1), sonicated for 3 min and kept at 60 °C for 30 min; (3) rinsed 10 times with distilled water; and (4) were finally oven-dried at 70 °C for at least 3 h. Vinyl-silanized glass substrates were prepared to chemically fix the hydrogels. The RCA-cleaned round glass substrates were immersed in 5% (*v*/*v*) vinyltrimethoxysilane in toulene (Sigma-Aldrich, St. Louis, MO, USA, 235768) and shaken for 18 h at room temperature. Thereafter the glasses were sequentially rinsed with acetone, ethanol and distilled water, and dried at 140 °C for 1 h [23].

### 2.2. Hydrogel Preparation

Poly-acrylamide hydrogels were prepared by fixing the cross-linker ratio to 2% combining a 40% acrylamide (AAm, Carl Roth, 7748.1) and 2% bis-acrylamide solution (bAAm, Carl Roth, 3039.2) in water. The molarity of the hydrogels varied between $0.6\;\mathrm{mol}\cdot {\mathrm{kg}}^{-1}$ and $2.0\;\mathrm{mol}\cdot {\mathrm{kg}}^{-1}$. A stock solution of about 1 mL was freshly prepared (Table 1), of which 200 μL was combined with 10% ammonium peroxodisulfate (4.6 μL, APS, Sigma, A3678) and *N*,*N*,*N*′,*N*′-tetramethylethylenediamine (0.3 μL, TEMED, Carl Roth, 2367.3). For each hydrogel, a 20 μL portion of the mixed solution was sandwiched between a round, vinyl-silanized glass substrate and a square-shaped cover glass and kept for 30 min at RT [24]. After removing the cover glass, the hydrogel was successively soaked in water for at least 48 h to remove the residual chemicals.

### 2.3. Mechanical Testing

The Young’s modulus *E* of the hydrogels was measured by nanoindentation using an atomic force microscope (NanoWizard, JPK Instruments, Berlin, Germany). The hydrogels were indented by a spherical colloidal probe that was attached to a silicon–nitride cantilever with a nominal spring constant of 0.08 N/m (CP-PNP-BSG; R = 5 μm, Olympus Optical) and an approach speed of 1 μm/s. Each cantilever was calibrated, and the spring constant was determined by thermal noise measurement [25]. The measured force–indentation curves were analyzed by nonlinear least-squares fitting to the Hertz model [22,26,27] using a customized MATLAB (Mathworks) routine. The modified Hertz model equation for spherical indenter shapes to determine *E* was fitted as

(1) $$F=4ER^{1/2}\cdot {\left[3{\left(1-\nu \right)}^{2}\right]}^{-1}\cdot \delta^{3/2},$$

where *F* is the force applied to the indenter, ν=0.5 is Poisson’s ratio, and δ is the indentation depth [3,28]. The average Young’s modulus of 50 independent indentation sites at two 100×100 μm${}^{2}$ areas was quantified for each hydrogel to ensure statistical significance.

### 2.4. Height Determination

The height of the hydrogels was measured by autofluorescence signal observation using a laser scanning microscope (LSM 710, Carl Zeiss Microscopy GmbH, Jena, Germany) equipped with a 40× magnification objective lens (Plan-Neofluar 40×/1.3 DIC numerical aperture 0.60; Carl Zeiss Microscopy GmbH, Jena, Germany). The following setting was used: 75% laser intensity, 488 nm excitation wavelength, 509 nm to 740 nm emission bandwidth, a pinhole of 40 and a gain of 1015. The serial images were recorded with a spatial resolution of Δx = Δy = 2.768 μm (128 × 128 pixels, 8 bit) and Δz = 1.0 μm. For every molarity, at least two hydrogels with five positions were measured. The average intensity signal of each image was calculated and normalized to determine hydrogel height. Height was obtained by measuring the distance between the autofluorescence intensity peaks at the glass-to-gel and gel-to-medium interfaces. All data were analyzed with custom routines in MATLAB (R2018b; MathWorks, Natick, MA, USA).

### 2.5. Surface Functionalization

For functionalizing the surface of the hydrogels, the catecholamine L-DOPA (3,4-Dihydroxy-L-phenylalanin, Sigma-Aldrich; D9628) was used. L-DOPA was dissolved in freshly prepared TRIS buffer ( 10 mM, pH 10, Roth 4855.2) at a final concentration of 2 mg/mL for 30 min in the dark on a tube roller, and sterilized through a 0.2 μm filter (Filtropur S0.2, Sarstedt 83.1826.001) [29]. The hydrogels were washed with TRIS buffer, and 250 μL of the L-DOPA solution was added to each hydrogel. After 30 min of incubation in the dark (RT), the samples were washed two times with PBS to remove unbound L-DOPA. On the functionalized hydrogel, fibronectin (FN) from human plasma (Sigma, F2006), prepared with PBS, was applied at the desired concentration (Table 2) and allowed to react for at least 2 h at 37 °C [3,30]. Prior to cell seeding, the hydrogels were washed with cell culture medium to remove unbound excess fibronectin.

### 2.6. Cell Culture

Mouse myoblast cells (C2C12, <30 passages, Sigma-Aldrich) were cultured in Dulbecco’s modified Eagle’s medium (DMEM, D6046, Sigma-Aldrich) supplemented with 10% fetal bovine serum (F9665, Sigma-Aldrich) and 1% penicillin-streptomycin (P0781, Sigma-Aldrich). The cells were enzymatically digested using trypsin–EDTA solution (0.25%, T4049, Sigma) to detach them from the culture flask. Thereafter, the cells were counted and seeded depending on substrate properties with a cell density between $5\;{\mathrm{cells}/\mathrm{mm}}^{2}$ and $70\;{\mathrm{cells}/\mathrm{mm}}^{2}$ on the fibronectin-coated substrates 24 h before observation (F2006, Sigma-Aldrich). The cells were maintained in a humidified incubator at 37 °C and 5% CO${}_{2}$.

### 2.7. Image and Time-Lapse Acquisition

Phase-contrast images were obtained using an inverted microscope (Axiovert 200M; Zeiss, Carl Zeiss Microscopy GmbH, Jena, Germany) through a 20× magnification objective lens (LD ACHROPLAN 20×/0.40; Carl Zeiss Microscopy GmbH, Jena, Germany) and a CCD camera (Zeiss AxioCam MRm; Carl Zeiss Microscopy GmbH, Jena, Germany) at a resolution of Δx = Δy = 0.322 μm (Axiovision, v4.8; Carl Zeiss Microscopy GmbH, Jena, Germany). Time-lapsed, phase-contrast images were obtained using an inverted microscope (Zeiss Cell Observer Z1; Carl Zeiss Microscopy GmbH, Jena, Germany) through a 40× magnification objective lens (Plan-Neofluar 40×/0.6 Korr Ph2, Carl Zeiss Microscopy GmbH, Jena, Germany) and a CCD camera (Zeiss AxioCam 503 mono) at a resolution of Δx = Δy = 0.332 μm after 2×2 binning under physiological humidified conditions (37 °C, 5% CO${}_{2}$).

### 2.8. Fluorescence Staining

Cells were washed with PBS and fixed with 4% formaldehyde (Promega, Madison, WI, USA, 104003) in PBS for 20 min at RT and washed three times for 10 min in PBST (0.1% Tween 20, Carl Roth, 9127.1, in PBS). Phalloidin (200 U/mL in methanol, Alexa fluor 546, Invitrogen, Karlsruhe, Germany, A22283) was added to DAPI ( 1 μg/mL in PBST) solution (1:800), and 400 μL per sample was incubated in the dark for 1 h at RT. The samples were washed three times for 10 min in PBST and stored in PBS at 4 °C until observation on the next day.

### 2.9. Lifeact Transfection

Cells were plated on hydrogels with a density of 100 cells/cm${}^{2}$ and incubated in DMEM for 24 h before transfection. On the day of transfection, cells were washed with PBS and DMEM was changed to antibiotic-free DMEM. For every transfection sample, 0.7 μL of plasmid (pCMV-Lifeact-TagGFP2, ibidi, 60101, 1 μg/μL) and 2.1 μL of Lipofectamine 2000 (Invitrogen, 11668-030) were added to individual microcentrifuge tubes containing 100 μL PBS each. Both tubes were then combined and incubated for 20 min at RT in the dark. The solution of about 200 μL was added dropwise to the cells while shaking the sample carefully. Medium was changed to DMEM containing penicillin–streptomycin 24 h after transfection, and cells were observed for 30 h.

### 2.10. Image Acquisition of Fluorescent Cells

DAPI- and phalloidin-stained C2C12 cells were imaged using the AxioObserver SD confocal microscope (Zeiss) equipped with Yokogawa CSU-X1 spinning disk unit using a 40× objective (Plan-Apochromat 1.4 Oil DIC UV, Zeiss) with the Axiocam 503 Mono CCD camera (Carl Zeiss Microscopy GmbH, Jena, Germany) at a resolution of Δx = Δy = 0.227 μm. For image acquisition and analysis, ZEN blue v2.3 software (Carl Zeiss Microscopy GmbH, Jena, Germany) was used. For visualisation of the nuclei and F-Actin, 405 nm (DAPI) and 561 nm (phalloidin) diode lasers were used. Each image was recorded as a 5 × 5 tile composition with 5 focal heights (Δz = 1 μm). Lifeact-tagged C2C12 cells were imaged using the same imaging system (561 nm diode lasers), but with an EMCCD camera (Photometrix Evolve 512) at a resolution of Δx = Δy = 0.333 μm. Each image was recorded as a 3 × 3 tile composition with 5 focal heights (Δz = 1 μm) every Δt=15 min. Tiles were stitched using the “fuse tiles” and “correct shading” features of the ZEN 2.3 software, followed by creating an orthogonal projection of the maximum intensity by merging all recorded Z-stacks.

### 2.11. Actin Quantification Analysis (AQuA)

The actin filaments of C2C12 cells were quantified by actin filament orientation analysis using an algorithm written in MATLAB (R2021b; The MathWorks, Natick, MA, USA) [3,5,16,31]. The Laplacian filter

(2) $$\left[\begin{array}{ccc} 0 & -1 & 0\\ -1 & 4 & -1\\ 0 & -1 & 0 \end{array}\right]$$

and n=15 differently rotated anisotropic Gaussians with $\sigma_{x}=4\sigma_{y}$ were convoluted to elongated Laplace of Gaussian (eLoG) kernels. The kernels were applied to the original images (tile composition), and the maximum response of each pixel was calculated as

(3) $$I_{\max}\left(n,x,y\right)=\max\left[\mathrm{eLoG}\left(n\right)\times I\left(x,y\right)\right].$$

Thereafter, $I_{\max}$ was processed by the binarized original images using the Otsu’s thresholding method [32]. Connected fibers of the same rotational direction with less than 10 pixels were removed. The obtained actin fibers were colorized with a color scheme that corresponds to the local actin orientation angles, $\theta_{n}$.

Phalloidin-stained and Lifeact-tagged cells were analyzed with $\sigma_{x}^{\mathrm{Phal}}=1$ and $\sigma_{x}^{\mathrm{Life}}=1.5$ so that $\Delta x/\sigma_{x}$ was constant and therefore proportionally scaled to the respective camera resolution Δx.

## 3. Results

**Figure 1:**
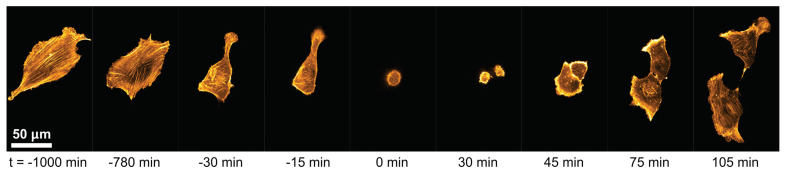
Proliferation dynamic of a single myocyte. Shown is an actin-tagged C2C12 muscle cell on glass that undergoes proliferation at about t=0 min. The two daughter cells increase their size and eventually migrate independently (t≥30 min).

Cell proliferation is a fundamental process for organismal development and homeostasis to ensure complete and precise cell duplication [33]. C2C12 myocytes follow a proliferation cycle of about 24 h when grown in a crowded environment. Figure 1 shows a typical example of a C2C12 cell that undergoes proliferation. The actin cytoskeleton is shown in order to highlight the dynamics and the sudden transition from the strongly adhered cell (t=−15 min) to the actin-depolymerized, rounded up cell just before cell division (t=0 min). Thereafter, the two daughter cells start to spread by increasing their actin polymerization activity. Once the cells overcome a critical size, they start to migrate again. This fundamental cell dynamic repeats and defines a cell cycle. The influence of extracellular matrix composition, i.e., substrate rigidity and protein density, is crucial for the cellular integrity of the cell cycle, as different mechanical stresses act on the cells and change their response. In this work, we statistically quantify dependencies and differences of cellular responses of single C2C12 cells on functionalized hydrogels.

**Figure 2:**
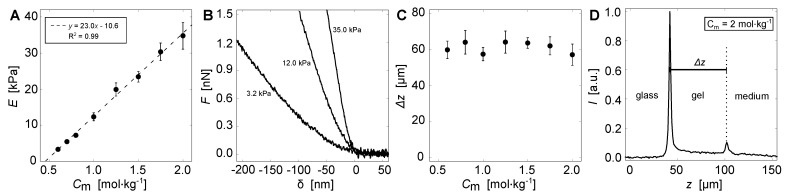
Property assessment of the poly-acrylamide hydrogels. (**A**) Average Young’s modulus *E* as a function of the total monomer concentration $C_{m}$ at fixed cross-linker ratio of 2%. The change in $C_{m}$ shows a linear increase in *E*, as illustrated by the dashed line. (**B**) Three exemplary nano-indentation curves measured at $C_{m}=0.6$, 1.0 and 2.0 mol·kg${}^{-1}$. Shown is force *F* as a function of indentation depth δ. (**C**) Average height Δz of the hydrogels as a function of $C_{m}$, obtained by auto-fluorescence measurements using confocal laser scanning microscopy. (**D**) Example of an auto-fluorescence signal of a hydrogel with $C_{m}$ = 2.0 mol·kg${}^{-1}$ (E=35 kPa). Shown is the normalized intensity as a function of height *z*. The two peaks mark the transitions from glass to gel and gel to culture medium, respectively. The gel height Δz is calculated as the peak-to-peak distance.

Polyacrylamide hydrogels were prepared as cell culture substrates to study dynamic changes to cell-morphology during cell growth and proliferation of single, spatially isolated C2C12 myocytes. The chemical composition of the hydrogel was adapted, i.e., total monomer concentration $C_{m}$ and cross-linker ratio, so that a single parametric dependence is sufficient to modulate *E*. Increases in $C_{m}$ lead to a monotonic decrease in the polymeric network mesh size at a fixed cross-linker ratio [34,35]. Contrarily, changes in the cross-linker ratio lead to non-monotonic changes in *E* when fixing $C_{m}$ [36] (see also Figure S1A), which may have additional effects on cell viability and dynamics [37]. Figure 2 illustrates the mechanical characterization of the prepared hydrogels. The increase in $C_{m}$ from 0.6 to 2.0 mol·kg${}^{-1}$ by fixing the cross-linker ratio to 2% led to a linear increase in *E* of about 3 to 35 kPa (Figure 2A, Table 3). This covers the biologically relevant elasticities in the microenvironment of C2C12 myocytes [14]. Figure 2B shows three exemplary nano-indentation curves for 3.2 kPa, 12.0 kPa and 35.0 kPa. Additionally, hydrogel heights were measured using auto-fluorescence signaling. No dependence on molarity was observed (Figure 2C). The average height of the hydrogels was measured to about Δz= 60 μm, which ensures that the cells cannot sense the underlying glass [24]. Figure 2D shows a typical example of an auto-fluorescence signal measured at a hydrogel with $C_{m}$ = 2.0 mol·kg${}^{-1}$. The two intensity peaks mark the transitions from glass to gel and gel to culture medium. While the relative height between both peaks can vary, the second peak (gel to medium) can be very shallow. In those cases, the shallow plateau was used to estimate gel height (Figure S1B).

### 3.1. Cell-Morphological Response to Substrate Properties

**Figure 3:**
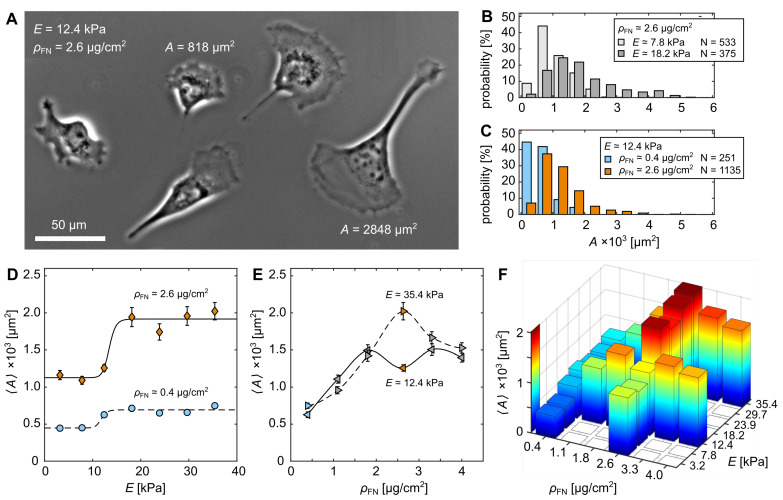
Cell area dependence on functionalized rigid hydrogels. (**A**) Phase contrast image of C2C12 cells on substrate rigidity E≃12.4 kPa and fibronectin coating density $\rho_{\mathrm{FN}}≃2.6$ $\mu {g/\mathrm{cm}}^{2}$. (**B**) Probability histograms of projected cell areas *A* for two substrate rigidities *E* at constant fibronectin density $\rho_{\mathrm{FN}}≃2.6$ $\mu {g/\mathrm{cm}}^{2}$. (**C**) Probability histograms of *A* for two different $\rho_{\mathrm{FN}}$ at constant E≃12.4 kPa. (**D**) Mean projected cell areas 〈A〉 as a function of *E* for two different $\rho_{\mathrm{FN}}$. The solid and dashed lines are fits of the Hill equation (Equation (4)). (**E**) 〈A〉 as a function of $\rho_{\mathrm{FN}}$ at E≃12.4 kPa (left pointed triangles) and E≃35.4 kPa (right pointed triangles). The solid and dashed lines are visual guidance. (**D**,**E**) Another visualization of the distributions is illustrated in Figure S2. The color scheme used in (**C**–**E**) relates to cells that are cultured on substrates functionalized with $\rho_{\mathrm{FN}}≃0.4$ $\mu {g/\mathrm{cm}}^{2}$ (blue) and $\rho_{\mathrm{FN}}≃2.6$ $\mu {g/\mathrm{cm}}^{2}$ (orange). (**F**) Two-dimensional histogram of 〈A〉 as a function of *E* and $\rho_{\mathrm{FN}}$. The error-bars indicate standard error (see Section 2).

The fibronectin-functionalized hydrogels were used to characterize the adhesion properties of C2C12 myocytes. A total of 13,426 cells with 22 different substrate conditions were observed, consisting of seven different substrate rigidities and six different fibronectin coating densities. Figure 3A illustrates C2C12 myocyte cells cultured on a hydrogel substrate with E≃12.4 kPa and $\rho_{\mathrm{FN}}≃2.6$ $\mu {g/\mathrm{cm}}^{2}$. The cells exhibit a broad variation in projected cell area *A* even when cultured on the same substrate. Figure 3B shows two representative histograms of *A* for cells cultured on substrates with rigidities E≃7.8 kPa and E≃18.2 kPa at a constant fibronectin density, $\rho_{\mathrm{FN}}≃2.6$ $\mu {g/\mathrm{cm}}^{2}$. As has been shown in various earlier studies [3,14,17,19], increased substrate rigidity leads to increased *A*. A similar tendency is observed when fixing substrate rigidity (E≃12.4 kPa) and varying $\rho_{\mathrm{FN}}$, as plotted for 0.4 $\mu {g/\mathrm{cm}}^{2}$ (blue) and 2.6 $\mu {g/\mathrm{cm}}^{2}$ (orange) (Figure 3C). An increase in $\rho_{\mathrm{FN}}$ leads to an increase in *A*. The relation between mean projected cell area 〈A〉 and substrate rigidity *E* for the two fibronectin surface-densities $\rho_{\mathrm{FN}}=0.4$ $\mu {g/\mathrm{cm}}^{2}$ (blue) and 2.6 $\mu {g/\mathrm{cm}}^{2}$ (orange) is plotted in Figure 3D. As all distributions are strongly skewed normal distributions, 〈A〉 was determined by a log-transformation using a Finney estimator (see Section 2) [38]. The obtained dependencies can be fitted by the empirical Hill equation (dashed lines)

(4) $$H\left(E\right)=\langle A\rangle =\frac{{\langle A\rangle }_{\max}-{\langle A\rangle }_{\min}}{{\left(E_{1/2}/E\right)}^{m}+1}+{\langle A\rangle }_{\min},$$

as demonstrated before [3,13,15], with typical characteristic half levels at $E_{1/2}≃11.8$ kPa and $E_{1/2}≃13.4$ kPa that represent natural soft muscle tissue. The cooperativity coefficient was arbitrarily fixed to m=20 because the data density does not allow a statistically significant estimation of the slopes between the minimum and maximum projected areas, ${\langle A\rangle }_{\min}$ and ${\langle A\rangle }_{\max}$. Further, we verified that an increase in fibronectin surface-densities $\rho_{\mathrm{FN}}$ leads not only to increased levels of ${\langle A\rangle }_{\min}$ and ${\langle A\rangle }_{\max}$ but also to an increase in $E_{1/2}$, considering $E_{1/2}$ is always about E≃12 kPa. Figure 3E shows 〈A〉 as a function of $\rho_{\mathrm{FN}}$ at the fixed substrate rigidities E≃12.4 kPa and E≃35.4 kPa. As expected, 〈A〉 increases for both conditions up to about $\rho_{\mathrm{FN}}≃2$ $\mu {g/\mathrm{cm}}^{2}$. While cells cultured on substrates with E≃12.4 kPa exhibits a slightly lower kink at $\rho_{\mathrm{FN}}≃2.6$ $\mu {g/\mathrm{cm}}^{2}$, a maximum 〈A〉 is observed for E≃35.4 kPa. This variation in 〈A〉 at $\rho_{\mathrm{FN}}≃2.6$ $\mu {g/\mathrm{cm}}^{2}$ can be explained by the sensitive response of cells around $E_{1/2}$. Small changes in *E* can have drastic changes in 〈A〉 (see E≃12.4 kPa, Figure 3D). An overview of all investigated experimental conditions is shown in Figure 3F.

**Figure 4:**
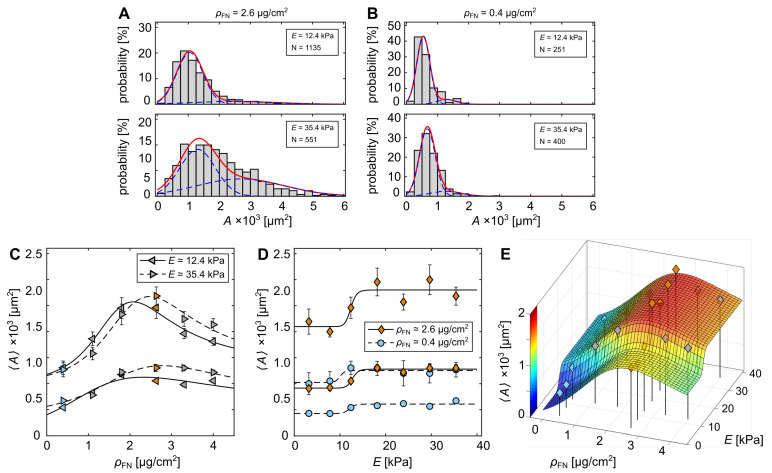
Cross-talk between hydrogel rigidity and fibronectin density. (**A**,**B**) Example of area distributions fitted by the mixture of two normal probability density functions (MN-pdf, see Supplemental Material). Red lines are MN-pdf, and blue lines are the two individual normal distributions. (**C**) Lower and higher means 〈A〉 of the obtained MN-pdfs as a function of $\rho_{\mathrm{FN}}$ at E≃12.4 kPa (left pointed triangles) and E≃35.4 kPa (right pointed triangles). Solid and dashed lines are fits of the Lorentz equation (Equation (5)) for the two Young’s moduli. The data correspond to the data shown in Figure 3E. (**D**) Lower and higher means 〈A〉 of the obtained MN-pdfs as a function of *E* for $\rho_{\mathrm{FN}}≃0.4$ $\mu {g/\mathrm{cm}}^{2}$ (blue circles) and $\rho_{\mathrm{FN}}≃2.6$ $\mu {g/\mathrm{cm}}^{2}$ (orange diamonds). Solid and dashed lines are fits of the Hill equation (Equation (4)). The error bars in (**C**,**D**) indicate the respective standard errors (see Supplemental Material). (**E**) Two-dimensional relationship of 〈A〉 as a function of *E* and $\rho_{\mathrm{FN}}$ corresponding to the data in (**C**,**D**). The fit was calculated by Equation (6).

An alternative explanation can be drawn from the skewed normal distributions (Figure 3C,D). The degree of skewness, i.e., right tail of distribution, strongly influences the resulting 〈A〉. Cells cultured on hydrogels with larger *E* and larger $\rho_{\mathrm{FN}}$ show stronger skewness. In order to take this effect into account and probe its effect on the statistical outcome, the area distributions were fitted to a mixture of two normal probability density functions (MN-pdf) (see Supplemental Material Equation (S10) and Figure 4A,B, solid red lines). This leads to two split 〈A〉 for each distribution, a lower and a higher one that represent the position of the first, main peak and shallower second peak for larger cells (see Figure 4A,B, blue dashed lines). In doing so, the degree of skewness loses its strong influence on the mean 〈A〉 calculated for the entire distribution. Figure 4C shows the two split 〈A〉 as a function of $\rho_{\mathrm{FN}}$ at the fixed substrate rigidities E≃12.4 kPa and E≃35.4 kPa. Following the approach of Zemel et al. (2010) for quantifying cell area as a function of substrate rigidity parameterized by the aspect ratio of cells [16], the data were fitted with the Lorentz-like function as

(5) $$L\left(\rho_{\mathrm{FN}}\right)=\langle A\rangle =\frac{x_{1}\rho_{\mathrm{FN}}}{x_{2}{\left(\rho_{\mathrm{FN}}-x_{3}\right)}^{2}+1}+x_{4},$$

where $x_{i}$ are fitting parameters. The black solid and dashed lines are the respective fits (Figure 4C). A clear correlation between 〈A〉 and $\rho_{\mathrm{FN}}$ is observed, although the degree of skewness, i.e., relative amount of larger cells, significantly varies between different substrate conditions. A maximum for all four curves is found at about 2 $\mu {g/\mathrm{cm}}^{2}$ to 3 $\mu {g/\mathrm{cm}}^{2}$. There is no slightly lower kink at $\rho_{\mathrm{FN}}≃2.6$ $\mu {g/\mathrm{cm}}^{2}$ visible (see Figure 3E for comparisons). Figure 4D illustrates 〈A〉 as a function of *E* following the same approach. The Hill fits lead also to comparable characteristic half levels at about $E_{1/2}≃12$ kPa and $E_{1/2}≃13$ kPa, as obtained in Figure 3D. Consequently, the kink found at $\rho_{\mathrm{FN}}≃2.6$ $\mu {g/\mathrm{cm}}^{2}$ can be explained by the lower number of cells that lead to the secondary normal distribution of larger cells. Thus, this can be interpreted as optimization of cellular mechanosensing to the ECM, as most of the cells have similar sizes, caused by the significantly lower degree of skewness (see also Figure 4A,B).

Taking advantage of the more-representative statistical analysis, all data were fitted to equation

(6) $$\langle A\rangle =\frac{H\left(E\right)+L\left(\rho_{\mathrm{FN}}\right)}{2},$$

where H and L correspond to the Hill equation (Equation (4)) and Lorentz-like equation (Equation (5)), respectively. Figure 4E shows the three-dimensional relation between the *E*, $\rho_{\mathrm{FN}}$ and 〈A〉. The latter is back-scaled by the fraction *p* between the two split 〈A〉 (see Supplemental Material Equation (S10)).

### 3.2. Cytoskeletal Actin Response to Substrate Properties

**Figure 5:**
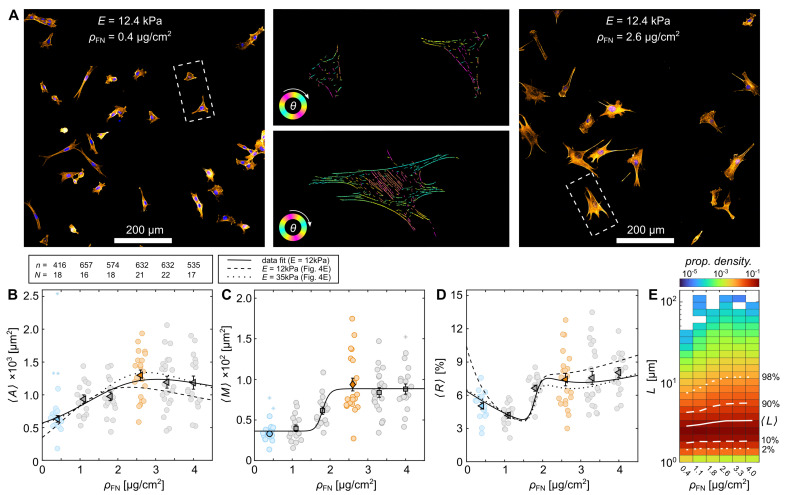
Actin cytoskeleton formation dependence on fibronectin density. (**A**) Actin- and cell-nuclei-labeled confocal images of C2C12 cells on E≃12.4 kPa hydrogels coated with $\rho_{\mathrm{FN}}≃0.4$ $\mu {g/\mathrm{cm}}^{2}$ (**left** panel) and $\rho_{\mathrm{FN}}≃2.6$ $\mu {g/\mathrm{cm}}^{2}$ (**right** panel). Two exemplary sections of prior-processed images by AQuA (**middle** panel) are illustrated from these two $\rho_{\mathrm{FN}}$ (dashed selection, **left** and **right** panel). (**B**–**D**) Statistical analysis of mean projected cell areas 〈A〉, mean amount of actin 〈M〉, and fraction of actin amount to cell area 〈R〉 of the 5×5 tiled images. The respective number of images *N* and sum of the containing cells, i.e., nuclei, *n* is written on top of (**B**). Each brightly colored round data point is calculated from one 5×5 tiled image, and the mean and standard error of those are shown by the darker-colored data points. Substrates functionalized with $\rho_{\mathrm{FN}}≃0.4$ $\mu {g/\mathrm{cm}}^{2}$ and $\rho_{\mathrm{FN}}≃2.6$ $\mu {g/\mathrm{cm}}^{2}$ are highlighted in blue and orange. Asterisks depict outliers (see Section 2). (**E**) Probability density of actin fiber length *L*. Percentiles of 2%, 10%, 90% and 98% are marked by dashed and dotted white lines. 〈L〉 is marked as a solid white line.

Next, we investigated F-actin formation in C2C12 muscle cells. After 24 h incubation, the actin and nuclei of cells were stained and observed with confocal microscopy (see Material and Methods). Figure 5A illustrates a snapshot of such an observation at two different fibronectin surface densities, $\rho_{\mathrm{FN}}≃0.4$ $\mu {g/\mathrm{cm}}^{2}$ and $\rho_{\mathrm{FN}}≃2.6$ $\mu {g/\mathrm{cm}}^{2}$, with E≃12.4 kPa. The two center panels correspond to sections of prior-processed images by AQuA (dashed rectangles left and right panel). The alignment angles of the actin filaments were visualized as a function of color scheme (see Section 2). No preferred alignment angle was observed, as homogeneous substrates without micro-grooves were prepared [5]. Statistical analysis of the average projected cell areas 〈A〉 and average amount of actin per C2C12 cell 〈M〉 revealed a similar Hill-function-like dependence on $\rho_{\mathrm{FN}}$ at fixed hydrogel rigidity E≃12.4 kPa (Figure 5B,C). 〈A〉 and 〈M〉 increased up to $\rho_{\mathrm{FN}}≃2.6$ $\mu {g/\mathrm{cm}}^{2}$ and were saturated thereafter. Contrary to the results shown in Figure 3E, here no kink at $\rho_{\mathrm{FN}}≃2.6$ $\mu {g/\mathrm{cm}}^{2}$ is observed, as the statistical approach differs. Here, a large field-of-view (about 1 × 1 mm${}^{2}$) was automatically analyzed without taking single cells into account. By scaling the integrated sum of cell area by the counted number of nuclei, we obtained an averaged ${\langle A\rangle }_{N}$ (Figure 3B, bright data points). This was used to calculate the mean 〈A〉. Again $L\left(\rho_{\mathrm{FN}}\right)$ fits the data well (Figure 5B, solid line). For comparison, the fit of L from Figure 4C (lower solid line) is plotted as a dashed line, which also represents the data well, as cells cultured on rigid hydrogels with $\rho_{\mathrm{FN}}≃2.6$ $\mu {g/\mathrm{cm}}^{2}$ and E≃12.4 kPa exhibit a more-pronounced normal distribution that is less skewed. The same statistical approach is used for 〈M〉 (Figure 5C). Contrarily, 〈M〉 follows a Hill-like distribution, as shown by the fit of $H\left(\rho_{\mathrm{FN}}\right)$ (Figure 5C), with a characteristic half level at about $\rho_{\mathrm{FN}}≃1.8$ $\mu {g/\mathrm{cm}}^{2}$. The fraction of actin amount to cell area 〈R〉 (see Equation (S3) in Supplemental Material) shows two distinct plateaus (Figure 4D). Both fits (solid and dashed line) derived from 〈A〉 and 〈M〉 are in good agreement with the data (triangles). A plateau at about ${\langle R\rangle }_{\max}≃7.5\%$ is observed from about 2 $\mu {g/\mathrm{cm}}^{2}$. Considering that the thickness of actin bundles is not directly taken into account in AQuA, this indicates the actin cytoskeleton is almost twice as dense when $\rho_{\mathrm{FN}}$ is at least 2.6 $\mu {g/\mathrm{cm}}^{2}$. This is also visible at the difference of actin fiber length *L*, as shown as a probability density distribution in Figure 5E. The average actin fiber length 〈L〉 is shown as a solid white stripe for all conditions, indicating a proportional relation to 〈A〉.

**Figure 6:**
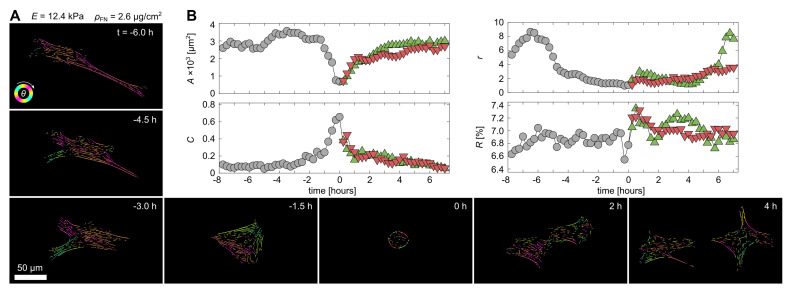
Proliferation dynamic of a single myocyte on a rigid hydrogel. (**A**) Snapshots of a C2C12 muscle cell that undergoes proliferation at about t=0 min on a E≃12.4 kPa hydrogel coated with $\rho_{\mathrm{FN}}≃2.6$ $\mu {g/\mathrm{cm}}^{2}$. The images were processed by AQuA. (**B**) Temporal tracking of the projected cell area *A*, the circularity *C*, aspect ratio *r* and fraction of actin amount to cell area *R* during 15 h of cell migration and proliferation. The mother and two daughter cells are distinguished by gray circles, green upward- and red downward-pointing triangles, respectively.

Monitoring actin fiber formation dynamics of single myocytes before and after proliferation shows similar results (Figure 6A). Here, on a hydrogel with $\rho_{\mathrm{FN}}≃2.6$ and E≃12.4 kPa, the area of cells *A* decreases only temporally before and after proliferation from a large, adhered cell, e.g., t=−6 h, to a round, barely adhered cell just before cell division at t=0 h, and back to large, adherent daughter cells after a few hours. Figure 6B shows the projected area *A*, the circularity *C*, aspect ratio $r_{A}$ and fraction of actin amount to cell area *R*. Once the mother cell (grey round points) divides into two daughter cells (green upward- and red downward-pointing triangles), all cell parameters recover within a few hours. The shape of the cell, e.g., *r*, changed rather dynamically during the observation time, while *A* and *C* showed rather stable plateaus before and after cell division. However, those plateaus are marked by the symmetric decrease and increase in *A* and *C*, respectively, since the proliferation of single cells in culture is marked by actin depolarization and thus rounding of the projected cell (t≃0, Figure 6B), similar to monitored cells that dynamically adjust to a softer ECM [3]. Contrarily, *R* does not change much during proliferation and seems therefore a stable cell-specific parameter. For this particular cell, *R* is about 7%.

In the next section, we investigated proliferation dynamics, e.g., temporal change in *A*, on rigid hydrogels with unlabeled cells to gain information on different substrate properties, as the fluorescence expression rate in life-imaging observations is rather unequally distributed among cells and might lead to phenotype-biased statistical results even in fluorescence-activated cell sorted (FACS) cultures (not shown).

### 3.3. Quantification of Proliferation Dynamics

**Figure 7:**
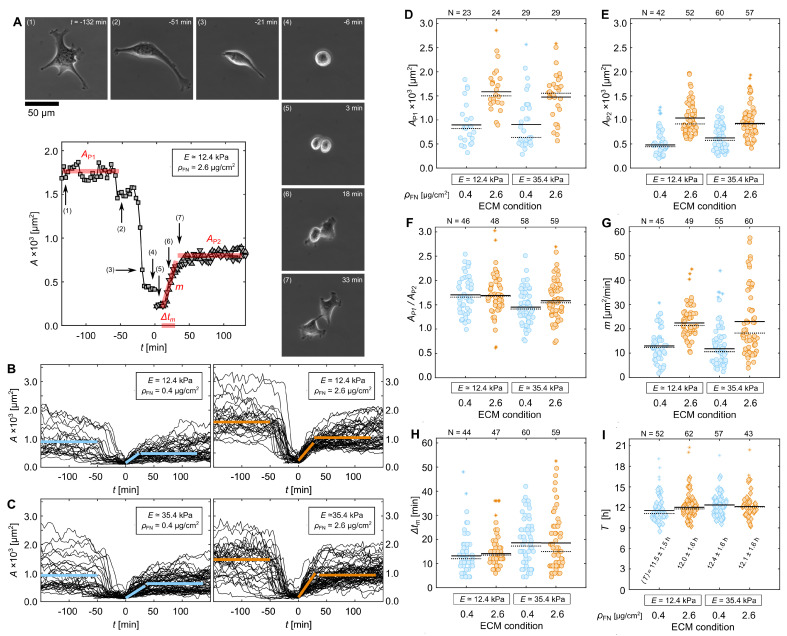
Proliferation dynamics depend on the ECM. (**A**) Example of a C2C12 muscle cell on a E≃12.4 kPa hydrogel coated with $\rho_{\mathrm{FN}}≃2.6$ $\mu {g/\mathrm{cm}}^{2}$, and the corresponding projected cell area *A* as a function of time. (**B**) The time evolution of the *A* of cells cultured on E≃12.4 kPa hydrogels coated with either 0.4 $\mu {g/\mathrm{cm}}^{2}$ (**left** panel) or 2.6 $\mu {g/\mathrm{cm}}^{2}$ (**right** panel). (**C**) The time evolution of the *A* of cells cultured on E≃35.4 kPa hydrogels coated with either 0.4 $\mu {g/\mathrm{cm}}^{2}$ (**left** panel) or 2.6 $\mu {g/\mathrm{cm}}^{2}$ (**right** panel). (**D**–**H**) Statistical analysis of the cell trajectories (**B**,**C**). Shown are the following extracted parameters: area plateau of the mother cells $A_{P1}$, area plateau of the daughter cells $A_{P2}$, area ratio of mother to daughter cells $A_{P2}/A_{P2}$, initial spreading rate *m* after cell division, and duration of spreading $\Delta t_{m}$ as a function of *E* and $\rho_{\mathrm{FN}}$. The exemplary parameters are highlighted in solid red lines in (**A**). (**I**) Cell cycle duration *T*, i.e., duration between two successive cell divisions. Means and medians are illustrated by solid and dotted lines, respectively. Number of data points is written on top of the plots as *N*, excluding outliers that are highlighted by asterisks. Substrates functionalized with $\rho_{\mathrm{FN}}≃0.4$ $\mu {g/\mathrm{cm}}^{2}$ and $\rho_{\mathrm{FN}}≃2.6$ $\mu {g/\mathrm{cm}}^{2}$ are highlighted in blue and orange, respectively.

Investigating temporal-resolved cellular dynamics offers a closer look at the dynamic responses to different substrate conditions. As it is unclear at what stage in the cell cycle each cell is during observation, we focus on proliferation dynamics, and therefore enable comparability of morphological changes. For single-cell observations, proliferation is clearly visible due to depolymerization of the actin cytoskeleton before the mother cell divides into two daughter cells (t = [−1:0] h, Figure 6). Further, in unlabeled cells this rapid decline can be observed in the change of cell area. A typical change in *A* is also observed after cell division as a rapid increase and subsequent initial plateau in *A* (Figure 7A). For statistical comparison, the different states were quantified by area plateau $A_{P1}$ before proliferation, the slope *m* and duration $\Delta t_{m}$, which describe linear cell spreading after proliferation, and the following area plateau $A_{P2}$ (red lines, Figure 7A). $A_{P1}$ was chosen to be at least 50 min before proliferation, as some cells exhibit a step-like decline in *A* followed by a sudden exponential decrease (see (2) and (3), Figure 7A). Both plateaus, $A_{P1}$ and $A_{P2}$, were defined at about, but not longer than, 100 min. Figure 7B,C show the projected area of all cells cultured on E≃12.4 and E≃35.4 kPa rigid hydrogels with two differently functionalized, low ($\rho_{\mathrm{FN}}≃0.4$ $\mu {g/\mathrm{cm}}^{2}$) and high ($\rho_{\mathrm{FN}}≃2.6$ $\mu {g/\mathrm{cm}}^{2}$) fibronectin concentrations. While a general substrate-conditional trend is visible, large variations among cells are observed within each condition, as seen by the quantified state parameter (Figure 7A–H). Both plateaus, $A_{P1}$ and $A_{P2}$, show hydrogel-rigidity independent mean values that vary only by the difference in $\rho_{\mathrm{FN}}$ (see for comparison Figure 3E, Figure 4C and Figure 5B). However, the fraction $A_{P1}/A_{P2}$ illustrates more stable recovery when cells are cultured on E≃12.4 kPa rigid hydrogels, as $A_{P1}/A_{P2}$ is larger and independent of $\rho_{\mathrm{FN}}$. The differences in area fraction are mainly governed by different cell spreading dynamics after proliferation. While *m* is again substrate-rigidity independent, $\Delta t_{m}$ depends on rigidity but surprisingly does not influence the amount of fibronectin. That means that cells cultured on stiffer hydrogels spread longer before reaching the plateau, but not faster, as *m* is comparable for both substrate rigidities. Further, increased variability among cells in *m* and $\Delta t_{m}$ is observed for stiffer hydrogels with a higher fibronectin condition (E≃35.4 kPa; $\rho_{\mathrm{FN}}≃2.6$ $\mu {g/\mathrm{cm}}^{2}$). The area plateaus, however, did not show such increased variability. To see how differences in spreading dynamics affect cell cycle duration *T*, independent time-lapse experiments were performed where only the cell division duration between two successive cell divisions was extracted. Although cells behave substantially differently with different substrate conditions (Figure 7A–H), no significant difference in *T* is observed. A mean cell-cycle duration 〈T〉 of about 12 h was observed (Figure 7I). As all cells were cultured 24 h prior to observation, we can estimate that the measured individual cell cycle duration corresponds to at least the third generation of cells.

## 4. Discussion and Conclusions

In this study, we explored the morphological response of single C2C12 cells to different compositions of substrate rigidity and fibronectin coating density. While a morphological transition from smaller to larger cells at about 12 kPa rigid hydrogels was observed, an optimal fibronectin density was identified ($\rho_{\mathrm{FN}}≃2.6\;\mu {g/\mathrm{cm}}^{2}$), where cells showed larger projected areas. The morphological transition showed a small increase of about 1.5 kPa from about 11.0 kPa at 0.4 $\mu {g/\mathrm{cm}}^{2}$ to about 12.5 kPa at 2.6 $\mu {g/\mathrm{cm}}^{2}$ (Figure 4D). We refrain from drawing conclusions and interpreting the observed trend, as only two FN coating densities were screened. However, we believe that the difference is related to the efficiency of cells in building the nanoscale architecture of integrin-based cell adhesions that may vary for different protein surface densities [39].

Variations in protein surface density could change the elastic properties of surfaces, and thus affect the morphology and function of cells. In this study, a fibronectin coating density of up to 4.0 $\mu {g/\mathrm{cm}}^{2}$ was used that corresponds to about 14,560 fibronectin $\mathrm{molecules}/\mu m^{2}$ (Table 2; see Supplemental Material) [40]. Considering an isotropic surface distribution of those FN molecules, since fibronectin coating is independent of hydrogel rigidity [41], a protein equilibrium distance (PED) of about 5 nm can be estimated (see Supplemental Material). The optimal fibronectin density was identified as about 2.6 $\mu {g/\mathrm{cm}}^{2}$, which corresponds to a PED of about 7 nm. This distance is in the order of the equilibrium distance of integrin in migratory cells, such as in mouse embryonic fibroblasts, with about 32 nm at the edge and 42 nm at the inner plasma membrane [39], and therefore reflects the nanoscale architecture of integrin-based cell adhesions. This result indicates that the optimum protein surface density is in the order of the natural focal adhesion density, as cells naturally grow in tissue surrounded by other cells of the same type. A similar optimized state is observed for the rigidity of the ECM at about 12 kPa, corresponding to the rigidity of muscle cells [2,14,15,16].

Rigidity and protein surface coating density also play major roles during cell proliferation. The duration of cell spreading correlates to the elastic modulus of the hydrogel, while spreading speed correlates to fibronectin density until reaching the area plateau. Thus, the lower the PED, the faster cells can find and attach to new adhesion sites at the edge of the plasma membrane. Although we did not observe any statistically significant differences in the cycle duration, these findings may also in part be explainable by deformation of the nucleus due to increased actomyosin tension, as previously reported [42]. Correlation between cell spreading and substrate elasticity has been shown before [43] and explained through substrate stress relaxation mediated through integrin adhesions and actomyosin-based contractility, and the increased nuclear translocation of Yes-associated protein (YAP)) driven by stress relaxation [44]. YAP is a transcriptional regulator that requires Rho activity to regulate mechanical properties of the ECM and cell geometry [45]. In mesenchymal stem cells, the percentage of cells with YAP in the nucleus increases linearly with increases in the elastic modulus, reaching a plateau at 10 kPa [46]. Considering a comparable YAP dependence in C2C12 cells, our findings suggest a potential link between cell spreading duration after proliferation with the content of YAP in the nucleus (Figure 7H), while cell spreading speed is solely regulated by the amount of matrix proteins in the ECM (Figure 7G). The exact mechanism of how the actin cytoskeleton impacts YAP in the context of mechanotransduction is still unknown [47]; however, YAP activation is known to increase cardiomyocyte proliferation and regeneration [48,49], which relates to our findings.

While both cell area and actin amount are good markers to identify the optimal fibronectin coating density, the presented results suggest that the amount of actin is the favorable cell characteristic to study the influence of extracellular matrix proteins. Another advantage is faster and more accurate automated data analysis, which was not reliably implementable for recorded phase-contrast cells. In this study, those images were manually segmented, as automated segmentation methods could not reach the quality necessary for comprehensive statistical analysis. Furthermore, the use of L-DOPA turned out to be much more reliable to functionalize matrix proteins to rigid hydrogels. Most studies use sulfo–SANPAH to functionalize hydrogels [3,42,50,51]; however, it is rather unreliable, and thus, difficult to reproduce comparable results. L-DOPA, on the other hand, has many advantages, such as a simpler protocol, no UV radiation, RT storage, relatively low price and artifact-free during imaging. Moreover, the investigation and comparison of other matrix proteins, such as collagen and laminin, in combination with L-DOPA would be useful to compare the influence of cell adhesion on fate and function in future studies.

In this study, we focused on the mechano-regulative feedback of single myocytes to different extracellular matrix properties. It has been shown before that ECM rigidity can critically influence cell fate and cell function. It remains to be shown whether similar influences can be correlated to matrix protein densities. The critical combination of both properties, however, is crucial to maintain cellular integrity and functionality, as we have shown in this study. More sensitive myocyte cultures, such as embryonic and inducible pluripotent stem cells [20], as well as primary cultures of neonatal animals [2], may be affected even stronger, and therefore, might be responsible for the life-threatening induction of alternans, where mechanoregulation, tissue arrangement and synchonization dynamics play an essential role [2,52,53]. The methodology and results presented in this manuscript provide a fundamental platform to investigate more complex myocyte dynamics, such as the proliferation dynamics of cells in crowded cell environments and differentiation dynamics of pluripotent cells in organoids, as cell shape may be stabilized by the tight junctions of neighboring cells and therefore also influence actin depolymerization dynamics.

## Figures and Tables

**Table 1:** Hydrogel preparation.

Total Monomer Concentration, $C_{m}$	H${}_{2}$O	AAm	bAAm
(mol·kg${}^{-1}$)	(μL)	(μL)	(μL)
0.60	780.2	104.5	92.5
0.80	714.5	139.3	123.3
1.00	648.9	174.2	154.2
1.25	566.8	217.7	192.7
1.50	484.7	261.2	231.3
1.75	402.6	304.8	269.8
2.00	320.6	348.3	308.3

**Table 2:** Protocol for fibronectin coating densities.

FN Coating Density, $\rho_{\mathrm{FN}}$	Volume on Gel	FN Concentration	FN Coating Density	Protein Equilibrium Distance
(μg/cm${}^{2}$)	(μL)	(μg/mL)	($\mathrm{molecules}/\mu m^{2}$)	(nm)
0.4	300	5.00	1456	17.2
1.1	250	16.72	4005	10.4
1.8	250	27.36	6554	8.1
2.6	250	40.00	9467	6.8
3.3	250	50.16	12,016	6.0
4.0	250	60.80	14,565	5.5

**Table 3:** Young’s modulus of hydrogels measured via AFM and linear fit (see Figure 2A).

Total Monomer Concentration, $C_{m}$	Young’s Modulus, *E* (Measured via AFM)	Young’s Modulus, *E* (Linear Fit)
(mol·kg${}^{-1}$)	(kPa)	(kPa)
0.60	3.3 ± 0.2	3.2
0.80	7.2 ± 0.2	7.8
1.00	12.3 ± 1.1	12.4
1.25	19.9 ± 1.8	18.2
1.50	23.4 ± 1.5	23.9
1.75	30.3 ± 2.5	29.7
2.00	34.8 ± 3.7	35.4

## Data Availability

The data that support the findings of this study are available on request from the corresponding authors.

## Supplementary Materials

The following are available online at https://www.mdpi.com/article/10.3390/cells11132122/s1, Figure S1: Property assessment of the poly-acrylamide hydrogels; Figure S2: Cell area dependence on functionalized rigid hydrogels.

## Abbreviations

The following abbreviations are used in this manuscript:

FN	fibronectin
RT	room temperature
ECM	extracellular matrix
AQuA	actin quantification analysis
DMEM	Dulbecco’s modified Eagle’s medium
eLoG	elongated Laplace of Gaussian
PED	protein equilibrium distance
