# Supplementary material for: Optimization of Mechanosensitive Cross-Talk between Matrix Stiffness and Protein Density: Independent Matrix Properties Regulate Spreading Dynamics of Myocytes
Source: Cells. 2022 Jul 5;11(13):2122. doi: 10.3390/cells11132122 (PMC9265304; doi:10.3390/cells11132122)
Supplement: Supplementary file 1 [file cells-11-02122-s001.zip › cells-1779530-supplementary.pdf]

SUPPLEMENTAL MATERIAL

Optimization of Mechanosensitive Cross-talk between Matrix Stiffness and Protein Density: Independent Matrix Properties Regulate Spreading Dynamics of Myocytes

Judith Brock[1], Julia Erhardt[1], Stephan A. Eisler[2] and Hörning, Marcel[1,*]

[1] Biobased Materials Laboratory, Institute of Biomaterials and Biomolecular Systems, University of Stuttgart, 70569 Stuttgart, Germany
[2] Stuttgart Research Center Systems Biology (SRCSB), University of Stuttgart, 70569 Stuttgart, Germany
[*] corresponding Author

Statistical analysis of fluorescent cells

Actin- and nuclei-stained cells that were recorded and statistically analysed as a 5 × 5 tile composition. The average cell area $\langle A \rangle$ and the average amount of actin per cell $\langle M \rangle$ was calculated using AQuA, as

$$\langle A \rangle = \sum_{5\times 5} A \times n_{\mathrm{nuc}}^{-1}, \tag{S1}$$

and

$$\langle M \rangle = \sum_{5\times 5} M \times n_{\mathrm{nuc}}^{-1} \tag{S2}$$

where n_{nuc} is the number of nuclei per image-composition, and $\langle M \rangle$ was obtained by AQuA. The fraction of actin amount to cell area for each image-composition was calculated, as

$$\langle R \rangle = \langle M \rangle \times \langle A \rangle^{-1}. \tag{S3}$$

Statistical analysis of phase-contrast cells

The statistical analysis of the projected cell areas was calculated by a log-transformation, because of the strongly skewed normal distributions, as

$$x_{\mathrm{log}}^{i} = \log(x^{i}), \tag{S4}$$

the mean μ_{log} and standard deviation σ_{log} was calculated, as

$$\mu_{\mathrm{log}} = \frac{1}{N}\sum_{i=1}^{N} x_{\mathrm{log}}^{i} \tag{S5}$$

$$\sigma_{\mathrm{log}} = \sqrt{\frac{1}{N}\sum_{i=1}^{N}\left(x_{\mathrm{log}}^{i} - \mu_{\mathrm{log}}\right)^{2}}. \tag{S6}$$

and back-transformed to obtain the mean and variance in the normal space as

$$\mu = e^{\,\mu_{\mathrm{log}} + \sigma_{\mathrm{log}}^{2}/2} \tag{S7}$$

$$\sigma^{2} = \mu^{2}\left(e^{\sigma_{\mathrm{log}}^{2}} - 1\right). \tag{S8}$$

following the Finney estimator approach[1], where N is the number of cells. Based on that the standard error with a 95% confidence interval is defined as

$$SE = \frac{2\sigma}{\sqrt{N}}. \tag{S9}$$

As an alternative approach the projected cell area distribution was quantified as a mixture of two normal probability density functions (MN-pdf), which is defined by the fractional sum of two normal probability density functions, as

$$P_\Sigma(x|p,\mu_{1,2},\sigma_{1,2}) = p \cdot P(x|\mu_1,\sigma_1) + (1-p) \cdot P(x|\mu_2,\sigma_2), \tag{S10}$$

with

$$P(x|\mu,\sigma) = \frac{1}{\sigma\sqrt{2\pi}} \exp\left(\frac{-(x-\mu)^2}{2\sigma^2}\right), \tag{S11}$$

where p is the fractional parameter defined between zero and one. The means $\mu_{1,2}$ and the respective standard error of the MN-pdf with a 95% confidence interval are defined as

$$SE_1 = \frac{2\sigma_1}{\sqrt{pN}} \quad \text{and} \quad SE_2 = \frac{2\sigma_2}{\sqrt{(1-p)N}}, \tag{S12}$$

where N is the number of cells observed at a certain culture condition.

Estimation of the protein equilibrium distance

For quantitative comparison the protein equilibrium distance (PED) of fibronectin (FN) was calculated as

$$\text{PED} = \sqrt{\frac{4\tilde{\rho}_{\text{FN}}}{\sqrt{3}}} \tag{S13}$$

with the immobilized molecular surface density

$$\tilde{\rho}_{\text{FN}} = \rho_{\text{FN}} \times m^{-1} \times m_{\text{C}}^{-1} \times b_{\text{eff}} \tag{S14}$$

where ρ_{FN} is the fibronectin coating density (see Table 2, main manuscript), $m = 450$ kDa the molecular weight of FN, $m_{\text{C}} = 1.66 \times 10^{-27}$ kg $= 1$ Da that is defined as 1/12 of the mass of a carbon-12 atom, and $b_{\text{eff}} = 4 \times 6.8\%$ the binding efficiency of L-DOPA, which is about four times higher than the binding efficiency of SulfoSanpah[2,3].

References

1. Finney, D.J. On the Distribution of a Variate Whose Logarithm Is Normally Distributed. Supplement to the Journal of the Royal Statistical Society 1941, 7, 155–161. doi:10.2307/2983663.

2. Wouters, O.Y.; Ploeger, D.T.; van Putten, S.M.; Bank, R.A. 3,4-Dihydroxy-L-Phenylalanine as a Novel Covalent Linker of Extracellular Matrix Proteins to Polyacrylamide Hydrogels with a Tunable Stiffness. Tissue Engineering Part C: Methods 2016, 22, 91–101. doi:10.1089/ten.tec.2015.0312.

3. Rajagopalan, P.; Marganski, W.A.; Brown, X.Q.; Wong, J.Y. Direct Comparison of the Spread Area, Contractility, and Migration of Balb/c 3T3 Fibroblasts Adhered to Fibronectin- and RGD-Modified Substrata. Biophysical Journal 2004, 87, 2818–2827. doi:10.1529/biophysj.103.037218.

Figures

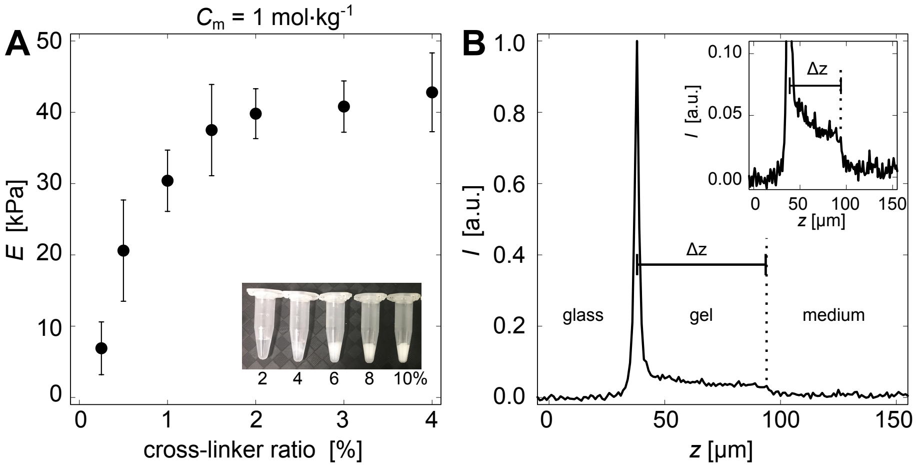

Figure S1 Property assessment of the poly-acrylamide hydrogels. **A** Mechanical property assessment of the poly-acrylamide hydrogels by the variation of the cross-linker ratio with a fixed total monomer concentration $C_m = 1$ mol·kg^{-1}. The inset depicts an image of poly-acrylamide hydrogels with different cross-linker ratios. Cross-linker ratios of 4% and higher lead to non-transparent hydrogels. **B** illustrates an example of the auto-fluorescence signal without a second peak (gel to medium) of a hydrogel with $C_m = 1.0$ mol·kg^{-}1 ($E = 12$ kPa). The inlet shows the shallow plateau that was used to estimate the gel height.

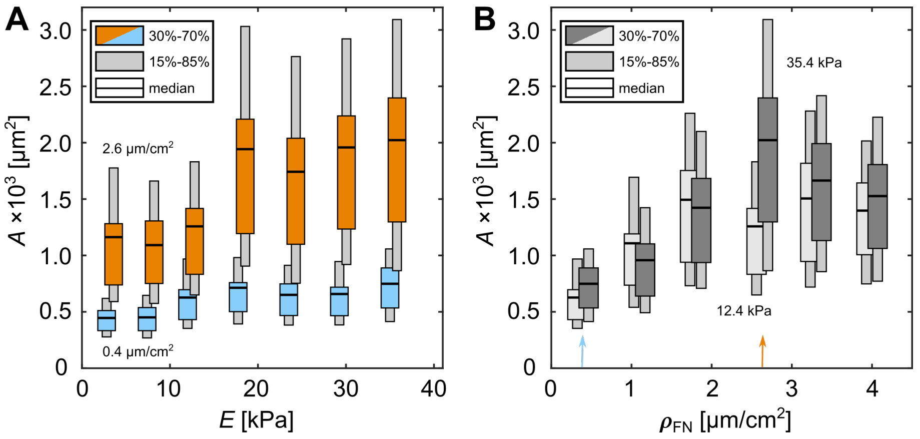

Figure S2 Cell area dependence on functionalized rigid hydrogels. **A** Projected cell areas, $\langle A \rangle$ as a function of E for $\rho_{\mathrm{FN}} \simeq 0.4$ µg/cm^2 (blue) and $\rho_{\mathrm{FN}} \simeq 2.6$ µg/cm^2 (orange). **B** $\langle A \rangle$ as a function of ρ_{FN} at $E \simeq 12.4$ kPa (bright grey) and $E \simeq 35.4$ kPa (dark grey). Shown are the median and percentiles of the cell area distributions (see Fig. 3**D-E**, main manuscript).
